# Meningitis

**Published:** 1842-07-16

**Authors:** 


### CLINICAL REPORTS.

*Blockley Hospital—Service of W. W. Gerhard, M. D.*

Reported by M. W. Wilson, M. D., Resident Physician.

*Cerebral Affections.*

*Meningitis.*—There was but one case of this, which occurred in J. T., a robust man, of middle age, who occasionally indulged immoderately in spirituous drinks. He had had several previous attacks, according to his own account, which, like the present one, were preceded by a "frolic;" (in other words, a fit of drunkenness.) In this instance, the disease manifested itself about two weeks after convalescence from delirium tremens. In the early part of the disease he complained of pain in the right and left hypochondria, and the abdomen was tumefied and tympanitic. The characteristic symptoms of meningitis soon followed; cephalalgia, distorted expression, frowning of the eye-brows, strabismus, contracted pupils, and delirium, with a corded not very frequent pulse. Under the use of the usual antiphlogistic remedies—bleeding, purging, mercurials, and cold applications to the head—the disease passed through a mild form, and the patient convalesced in about four weeks.

